# To lie or to tell the truth? The influence of processing the opponent’s feedback on the forthcoming choice

**DOI:** 10.3389/fpsyg.2024.1275884

**Published:** 2024-05-09

**Authors:** Maya Zheltyakova, Alexander Korotkov, Denis Cherednichenko, Michael Didur, Maxim Kireev

**Affiliations:** N.P. Bechtereva Institute of the Human Brain, Russian Academy of Science, Saint Petersburg, Russia

**Keywords:** deception, reward, feedback, decision-making, fMRI

## Abstract

**Introduction:**

The brain mechanisms of deceptive behavior are relatively well studied, and the key brain regions involved in its processing were established. At the same time, the brain mechanisms underlying the processes of preparation for deception are less known.

**Methods:**

We studied BOLD-signal changes during the presentation of the opponent’s feedback to a previous deceptive or honest action during the computer game. The goal of the game was to mislead the opponent either by means of deception or by means of telling the truth.

**Results:**

As a result, it was shown that several brain regions that were previously demonstrated as involved in deception execution, such as the left anterior cingulate cortex and anterior insula, also underlie processes related to deception preparation.

**Discussion:**

The results obtained also allowed us to suggest that brain regions responsible for performance monitoring, intention assessment, suppression of non-selected solutions, and reward processing could be involved in shaping future action selection and preparation for deception. By shedding light on the brain mechanisms underlying deception, our study contributes to a deeper understanding of this complex cognitive process. Furthermore, it emphasizes the significance of exploring brain mechanisms governing the choice between deception and truth at various stages of decision-making.

## 1 Introduction

The neurobiology of deceptive behavior has been relatively well studied, and key brain regions involved in its processing have been established due to extensive neuroimaging research. Most such studies aim to reveal brain activity, while subjects act dishonestly to manipulate opponents’ beliefs about the current situation. However, how previous behavior influences dishonest behavior remains under investigation, and only a handful of studies have tried to uncover brain activity associated with these processes. As with practically every type of behavior, deception is highly dependent on how current behavioral goals are achieved. In competitive settings, such as a game with an opponent, this goal is reduced to the goal of winning the game by maximizing the number of gains. When people resort to deception to obtain monetary rewards and avoid monetary punishments, acquiring information regarding the intermediate results of deception in terms of gains or losses becomes particularly relevant. In experimental settings, this information could be delivered by feedback from opponents signaling the effectiveness of the current deceptive behavior. In this case, the moment of receiving such feedback from opponents is the right time point to update the forthcoming behavior, given the strategic goal of the interaction. However, the brain mechanisms associated with such feedback processing coupled with consequent deceptive actions are practically not investigated. Therefore, studying the reward processing associated with the processing of deception extends the scope of its research toward a deeper understanding of its neurobiological basics.

In line with that, it was previously demonstrated that successful compared to unsuccessful outcomes of deception induced increased activity in the ventral striatum and medial orbitofrontal cortex (Sun et al., 2015). The loss compared to winning after lying resulted in a more negative-going ERP component called medial frontal negativity, which reflects the decrease of dopamine in the midbrain due to a less rewarding outcome (Hu et al., 2015). Although these results emphasize the importance of brain mechanisms of reward processing for deception, there are controversies in revealing specific characteristics of reward-associated brain mechanisms involved in processing the feedback received after lying compared to truth-telling. On the one hand, it was suggested that the internal cost of lying devalues the reward obtained due to deception, which manifested in decreased amplitude of the ERP component of reward positivity (Zhu et al., 2019). On the other hand, it was argued that successful dishonesty, compared to honest choices, causes increased positive outcome evaluation and attention processing by eliciting stronger activation in the ventral striatum and posterior cingulate cortex and a smaller feedback-related negativity ERP component (Sun et al., 2015). Both controversial suggestions emphasize that the preceding decision to deceive influences feedback processing, which manifests itself either as a modified amplitude of ERP components and/or as changes in activity in the areas of the brain referred to as reward processing. However, the brain mechanisms by which information about the outcome is processed in a way that influences the choice of forthcoming deceptive or honest actions are poorly studied.

In particular, this aspect of the relationship between feedback and deceptive action was investigated only by focusing on the question of how individual characteristics of reward processing were related to the tendency of subjects to deceive. It has been shown that the frequency of deception during the experiment was positively correlated with the psychological sensitivity to reward (Ding et al., 2013). Such dependencies were associated with the response to an anticipated reward in the nucleus accumbens (Abe and Greene, 2014), with the strength of the BOLD signal in the Brodmann area 10 associated with reward (Kelsen et al., 2020), and with the amplitude of the P300 component reflecting the size of the reward (Hu et al., 2015). Taken together, these data corroborate that people who are more sensitive to reward tend to lie more in the course of the experiment. To our knowledge, only one study directly compared deception and truth during the period prior to selecting a particular response (Ofen et al., 2017). During the preparation of the lie (vs. the preparation of truth), compared to the execution of the lie (vs. the execution of truth), separate regions in the superior parietal lobule were more active. Importantly, the design of the described study did not include feedback on deception, and participants received instructions on how to prepare to lie or tell the truth. This substantially deviates from the ecological validity of experimental deception, an issue widely discussed in the relevant literature dedicated to studying deception (Greene and Paxton, 2009; Sip et al., 2012; Kireev et al., 2013; Lisofsky et al., 2014). In summary, the influence of reward processing on the free choice to deceive by direct comparison between deception and truth has not yet been studied.

To fill this gap, the current study aims to define neural characteristics associated with feedback processing preceding the choice to deceive (vs. to tell the truth). Previously, it was shown that a stronger reaction to reward can increase the tendency to deceive. In line with this, we hypothesize that the activity in the reward-associated brain areas reflecting the reaction to the outcome will be increased when, in a forthcoming action, the subject decides to deceive, as compared to the forthcoming truth.

## 2 Materials and methods

### 2.1 Participants

A total of 24 volunteers (10 males and 14 females) were invited to participate in the experiment, with a mean age of 29.3 (SD = 6.5). All participants were right-handed, according to the Edinburgh Handedness Inventory (Oldfield, 1971), without any history of psychiatric and neurological diseases or current medication intake. All participants provided their written informed consent. All procedures were carried out in accordance with the Declaration of Helsinki and were approved by the Ethics Committee of the N.P. Bechtereva Institute of the Human Brain, St. Petersburg, Russia.

### 2.2 Stimuli and procedure

Data were obtained during the task procedure developed for the fMRI recording earlier (Kireev et al., 2013). Participants played the computed game, the goal of which was to mislead the opponent by means of deception or by means of telling the truth. More precisely, they saw an arrow facing upward or downward and had to inform the opponent about the direction of the arrow. After each statement, the opponent answered the participant whether he/she agreed or disagreed with this statement. The goal of the player was to make the opponent agree with the false statement and disagree with the truthful one. Conditions created by the current experimental task are related to real-life behaviors such as gambling. Therefore, the obtained results can be relevant in the field of psychiatry and medicine in order to improve the diagnosis and treatment of gambling disorders and addictions in general.

The participants were told that the role of the opponent was played by the computer, which obtained a specific mathematical algorithm to “create a model” of their behavior (sequences of choices) in order to effectively predict his or her actions (choices). Unknown to participants, feedback stimuli were automated to be presented in random order. The opponent’s feedback was predetermined, so the computer agreed to 60% of the trials to promote the use of deception. This distribution allowed approximately the same number of choices to be obtained to deceive and to tell the truth. It was chosen based on results previously obtained in the event-related potential study using the same task (Kireev et al., 2007).

The task consisted of game trials and additional control trials, in which participants were instructed to tell only the truth. Each trial began with the presentation of an arrow for 500 ms. The participants made the decision and sent the statement to the opponent by pressing the button during 4.5 s, starting with the arrow presentation. Pressing the button with the thumb corresponded to the statement ‘arrow faces up’, and using the index finger corresponded to the statement ‘arrow faces down’. When the sent direction coincided with the actual direction of an arrow, the trial was considered true, and when it was not, it was deceptive. Then, feedback was presented as follows: for 500 ms, the participants saw the opponent’s response (‘agree’ or ‘disagree’), and for 500 ms, the monetary consequence was presented. Successful trials (when the opponent agreed with deception and disagreed with truth) were rewarded with 2 or 5 rubles. Failed trials (when the opponent disagreed with deception and agreed with truth) were punished by subtracting 2 or 5 rubles from the player.

Control trials had the same temporal characteristics but were marked with different arrow colors. After sending the statement, participants saw the feedback—‘accepted’. They received no reward for providing the correct information in control trials, but when failing to follow the instruction and reporting the wrong arrow direction, they were punished by subtracting 2 rubles from the player.

Intertrial interval varied between 500 and 2,500 ms with 500 ms steps in a randomized manner.

Participants were instructed that monetary rewards and punishments were added to and subtracted from the final amount of money they received for participation in the study. They also knew that the monetary result varied across two experimental sessions. Each session lasted 15 min and consisted of 90 trials, including 60 free-choice game trials and 30 control trials. In one of the sessions, the consequence of false statements (both successful and unsuccessful) was equal to 5 rubles, and the consequence of truthful statements was equal to 2 rubles. The other session was characterized by the opposite ratio of monetary rewards and punishments for deception and truth. Therefore, the task design allowed manipulating monetary results, while participants were aware of their choice’s stakes for every trial.

### 2.3 fMRI image acquisition procedure and image processing

fMRI data were recorded using a 3 Tesla Philips Achieva scanner. Structural images were acquired using a T1-weighted pulse sequence (T1W-3D-FFE; repetition time [TR] = 2.5 ms; echo time [TE] = 3.1 ms; 30° flip angle), measuring 130 axial slices (field of view [FOV] = 240 × 240 mm; 256 × 256 scan matrix) of 1-mm thickness. Functional images were obtained using an echo planar imaging (EPI) sequence (TE = 35 ms; 90° flip angle; FOV = 208 × 208 mm; 128 × 128 scan matrix). In total, 32 continuous 3.5-mm-thick axial slices (voxel size = 3 × 3 × 3.5 mm) covering the entire cerebrum and most of the cerebellum were oriented with respect to structural images. The images were acquired using a TR of 2000 ms.

An MR-compatible cervical collar was used to prevent head movements. The presentation of stimuli, recording of participant responses, and synchronization with functional image acquisition were performed using the *In vivo* Eloquence fMRI System and Eprime 1.1. software (Psychology Software Tools Inc., Pittsburgh, PA, USA).

Data pre-processing and subsequent statistical analysis were performed in the SPM8 and SPM12 toolbox^1^ run in MATLAB (Mathworks Inc., Natick, MA, USA). Pre-processing of raw fMRI data for each participant included the following stages: realignment, slice-time correction, co-registration, normalization, and smoothing (8-mm FWHM). During the realignment stage, six parameters of head movement relative to the first image were generated (translations and rotations in three coordinate axes).

### 2.4 Statistical analysis

One participant was excluded from the statistical analysis because he did not follow the task and performed only truthful actions. The following procedure was applied to 23 participants.

First, general linear models (GLMs) were created for each participant separately. They included eight regressors representing temporal characteristics of experimental events. The first three modeled players’ decisions in deceptive, truthful, and control trials with the onset at the time of sending messages (button press) and duration equal to zero. Following four modeled feedback processing with the onset at the beginning of feedback presentation and duration equal to 1. Feedback stimuli were classified according to the result and the decision made in the following trial: victory followed by deception, victory followed by truth, defeat followed by deception, and defeat followed by truth. GLMs also included feedback on control trials and mistakes in one separate regressor of no interest and six regressors for six head movement parameters obtained during pre-processing (realignment) (Johnstone et al., 2006). The regressors were then convolved with the standard hemodynamic response function.

Second, the beta values of the regression coefficients for the regressors in GLMs were estimated at the individual level of analysis. Linear contrasts of the beta coefficients of each of the four feedback regressors and the baseline were calculated and used as a variable for the second-level analysis.

In the second-level random-effect analysis, the models included two factors with two levels: ‘result’ (victory or defeat) and ‘forthcoming action’ (deception or truth). The T-contrasts for the main effect of the result and the main effect of the forthcoming action were calculated.

Finally, the obtained T-contrasts were used to make a voxel-wise statistical inference at a group level. For estimation and making inferences, Bayesian inference (as implemented in SPM12) was used (Friston et al., 2002a,^26,25^; Neumann and Lohmann, 2003). This approach is more advantageous than the standard so-called frequentist statistics because it overcomes problems related to zero-hypothesis significance testing. Namely, it is not sensitive to the multiple comparisons problem and type-II errors appearing from necessary corrections. This method, based on Bayesian statistics, estimates the presence or absence of the effect of interest based on the calculation of posterior probability maps for contrasts of interest. In the context of the present study, the posterior probability refers to the probability of the difference between conditions (i.e., the contrasts obtained during the first-level analysis) being larger than zero. In the present study, the voxel-wise effect size threshold equal to 0 was used because no effect size threshold is necessary to apply the Bayesian approach to statistical inference (Thirion et al., 2007). A posterior probability map threshold defined as a log-odds threshold was applied to assess the significance of the main effects. A gray matter mask, created from segmented structural images, was used to select only voxels within the gray matter in all subjects. Xjview Toolbox^2^ was used to identify the anatomical location of the obtained clusters. MRIcroGL was used to visualize results and create illustrations^3^. The parcellation atlas of the right temporoparietal junction (TPJ) by Mars et al. (2012) was used to identify clusters located within the right TPJ and its anterior and posterior subdivisions.

## 3 Results

### 3.1 Behavioral results

The current study aims to define BOLD-signal changes associated with feedback processing preceding the choice to deceive (vs. to tell the truth). To support the fMRI-data analysis, we estimated the effect of feedback itself on behavioral characteristics to eliminate its influence on the obtained result. For each subject, the rate of forthcoming deception (vs. honesty) was calculated for four feedback types (successful deception, successful truth, unsuccessful deception, and unsuccessful truth) using the following formula: *Rate = (Next deception − Next truth)/(Next deception + Next truth).* No significant differences were found for the mean calculated rate between the four feedback types [Friedman ANOVA Chi Sqr. (3, 23) = 2.29, *p* > 0.05].

### 3.2 Observation of feedback preceding the choice to deceive and to tell the truth

**Figure 1:**
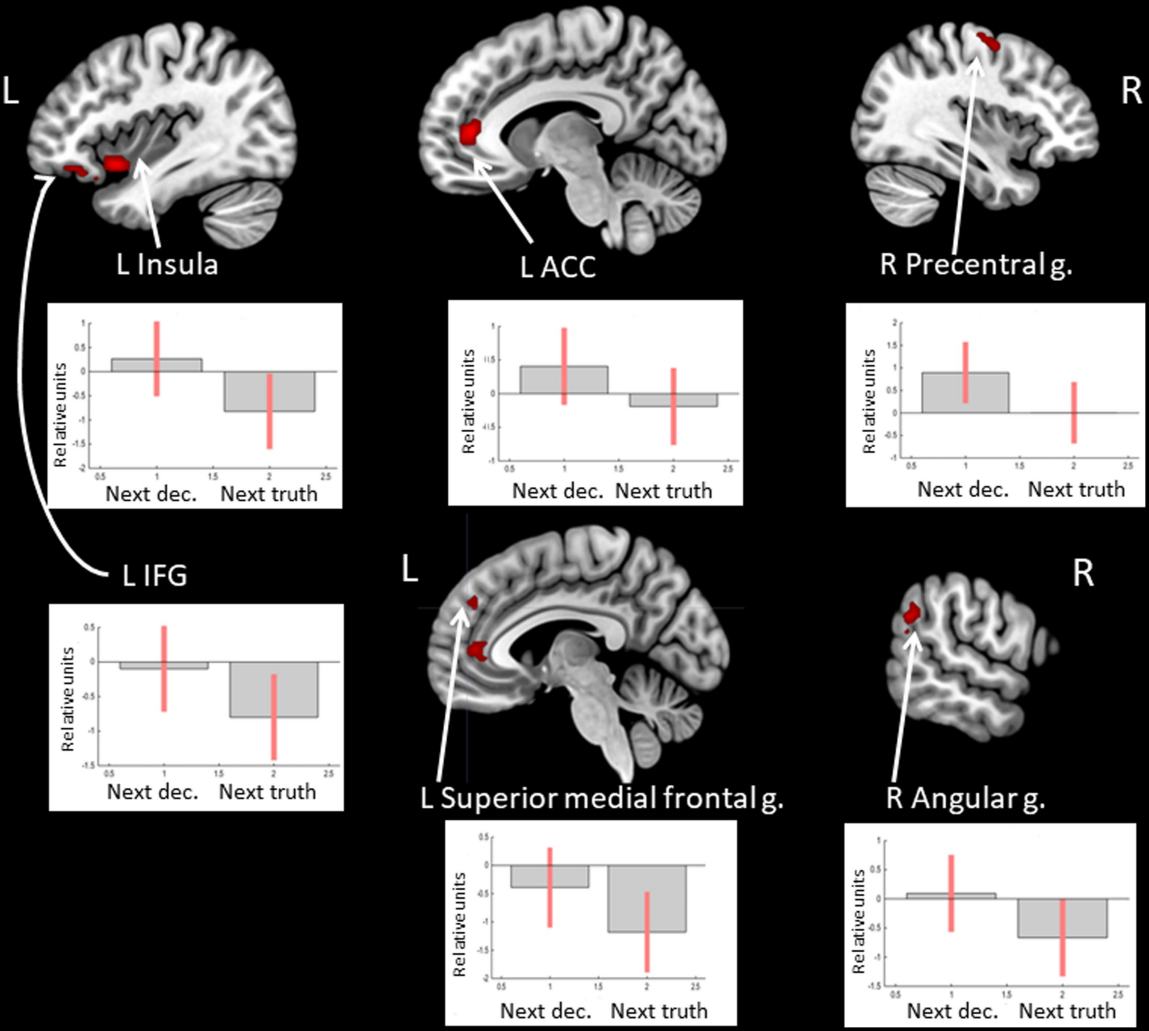
Clusters of increased BOLD signals associated with the observation of feedback preceding the deceptive compared to the truthful action are colored red (t-contrast ‘next deception> next truth’, log odds>5, *k* = 20). Plots show effect sizes for conditions of interest (vs. baseline) with 90% confidence intervals. R/L, right/left hemisphere; n., nucleus; g., gyrus; IFG, inferior frontal gyrus; ACC, anterior cingulate cortex; dec, deception.

Observation of feedback preceding the choice to deceive compared to the choice to tell the truth was associated with increased BOLD signals in the anterior cingulate cortex (ACC), the anterior insula, the inferior frontal gyrus (IFG), and the medial superior frontal gyrus in the left hemisphere and the precentral gyrus, the supramarginal gyrus, and the angular gyrus in the right hemisphere (see Figure 1 and Table 1). In particular, the beta values of the regression coefficients of the regressors of interest in the right angular gyrus, the left IFG, and the left medial superior frontal gyrus were negative, i.e., less negative for the feedback stimulus preceding deceptive actions (choices reported by pressing the button). Thus, in the named areas, increased suppression of local brain activity was observed prior to performing a truthful compared to deceitful action. The peak of the cluster in the right angular gyrus was also located in the right TPJ area, and the cluster overlapped with both its anterior and posterior subdivisions (Mars et al., 2012).

**Table 1:** Clusters of increased BOLD signals associated with the observation of feedback preceding the deceptive compared to the truthful action (t-contrast ‘next deception > next truth,’ log odds>5, *k* = 20).

**Brain region**	***k***	**Log odds**	**Peak MNI coordinates**		
			***x***	***y***	***z***
R Precentral g./ Middle frontal g.	25	9.33	33	−10	61
		6.78	39	−4	58
L ACC	38	9.16	−9	41	7
L Insula/Temporal pole	45	8.47	−39	11	−11
		5.94	−51	11	−29
L IFG/Insula	45	8.03	−42	35	−14
		6.36	−27	20	−17
		5.96	−33	26	−20
R Angular g./supramarginal g.	22	7.62	60	−55	31
L Superior medial frontal g.	27	6.45	−3	47	31
		5.47	3	47	19

R/L, right/left hemisphere; g., gyrus; IFG, inferior frontal gyrus; ACC, anterior cingulate cortex; k, cluster size in voxels.

**Figure 2:**
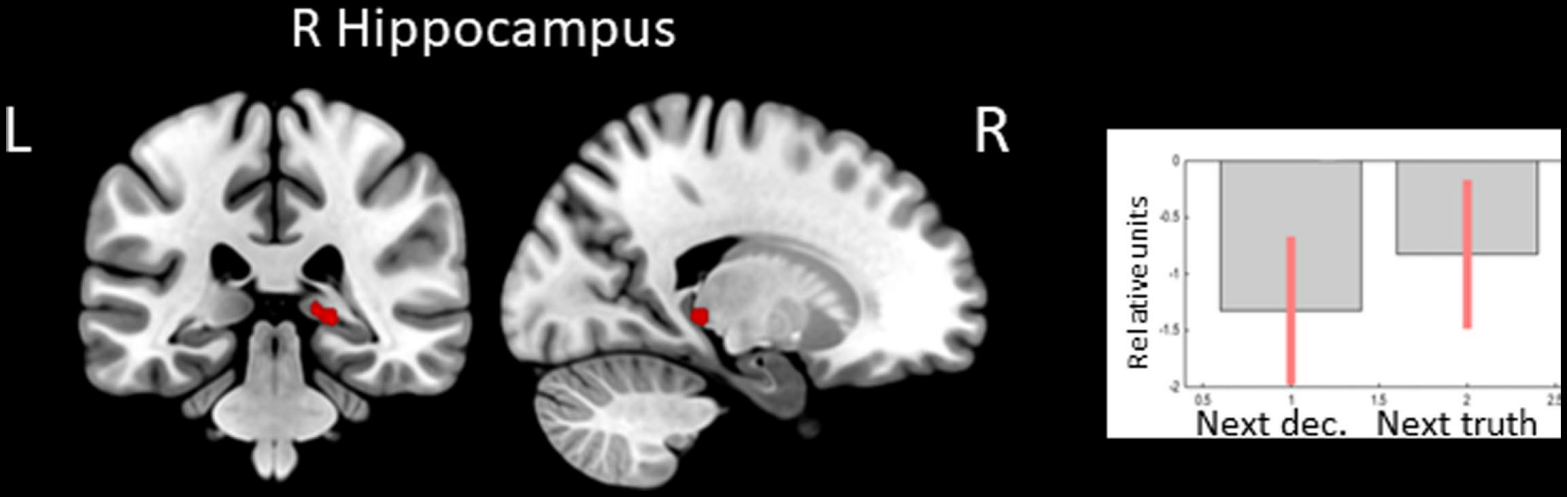
Clusters of increased BOLD signals associated with the observation of feedback preceding the honest compared to the deceptive action are colored red (t-contrast ‘next truth> next deception’, log odds>3, *k* = 10). Plots show effect sizes for conditions of interest (vs. baseline) with 90% confidence intervals. R/L, right/left hemisphere; dec, deception.

In the opposite contrast, observing feedback preceding the choice to tell the truth compared to the choice to lie was associated with less decreased BOLD signal in the right hippocampus (see Figure 2 and Table 2).

**Table 2:** Clusters of increased BOLD signals associated with the observation of feedback preceding the honest compared to the deceptive action (*t*-contrast ‘next truth > next deception,’ log odds>3, *k* = 10).

**Brain region**	***k***	**Log odds**	**Peak MNI coordinates**		
			***x***	***y***	***z***
R hippocampus	17	4.10	21	−34	−2

R/L, right/left hemisphere; k, cluster size in voxels.

### 3.3 Observation of feedback informing about victory or defeat

**Figure 3:**
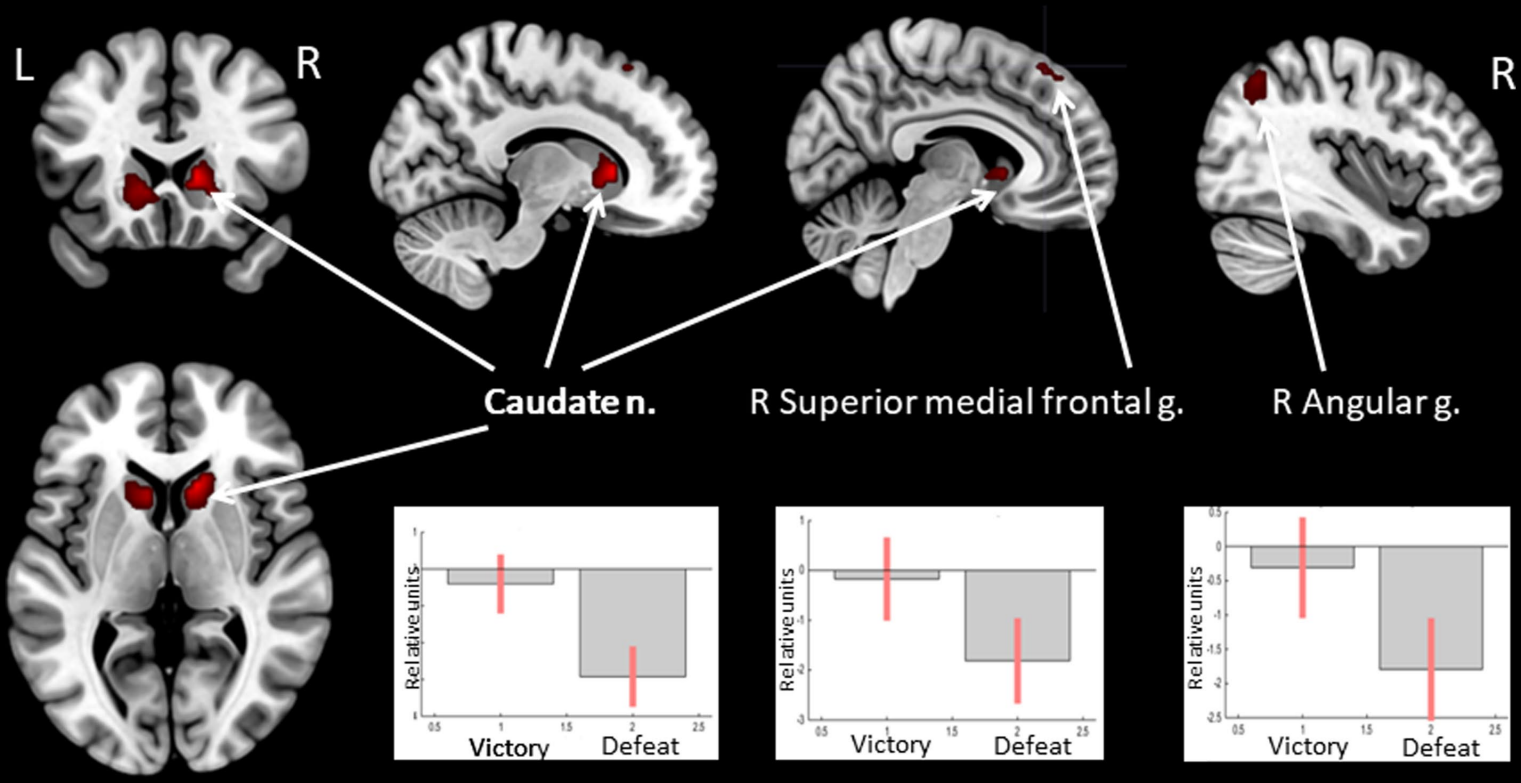
Results of the group-level BOLD-signal analysis. Clusters of increased BOLD signals associated with the observation of feedback informing about victory compared to defeat are colored red (t-contrast ‘victory> defeat’, log odds>10, *k* = 30). Plots show effect sizes for conditions of interest (vs. baseline) with 90% confidence intervals. R/L, right/left hemisphere; n., nucleus; g., gyrus.

Receiving of feedback denoting victory compared to defeat was associated with relatively higher BOLD signals in the caudate nucleus (CN) bilaterally, the right angular gyrus, and the superior medial frontal gyrus (see Figure 3 and Table 3). Considering that the beta values of the regression coefficients of the regressors of interest in these areas were negative, the result signified an increased suppression of activity when participants lost compared to when they won.

**Table 3:** Clusters of increased BOLD signals associated with the observation of feedback that means defeat compared to victory (*t*-contrast ‘victory> defeat’, log odds>10, *k* = 30).

**Brain region**	***k***	**Log odds**	**Peak MNI coordinates**		
			***x***	***y***	***z***
R/L caudate n.	204	35.35	12	20	4
		26.93	−9	17	1
		11.85	15	14	16
R angular g.	49	15.1	39	−61	49
R superior medial frontal g./superior frontal g.	38	13.82	6	35	55
		12.6	3	41	49
		12.42	18	23	58

R/L, right/left hemisphere; n., nucleus; g., gyrus; k, cluster size in voxels.

**Figure 4:**
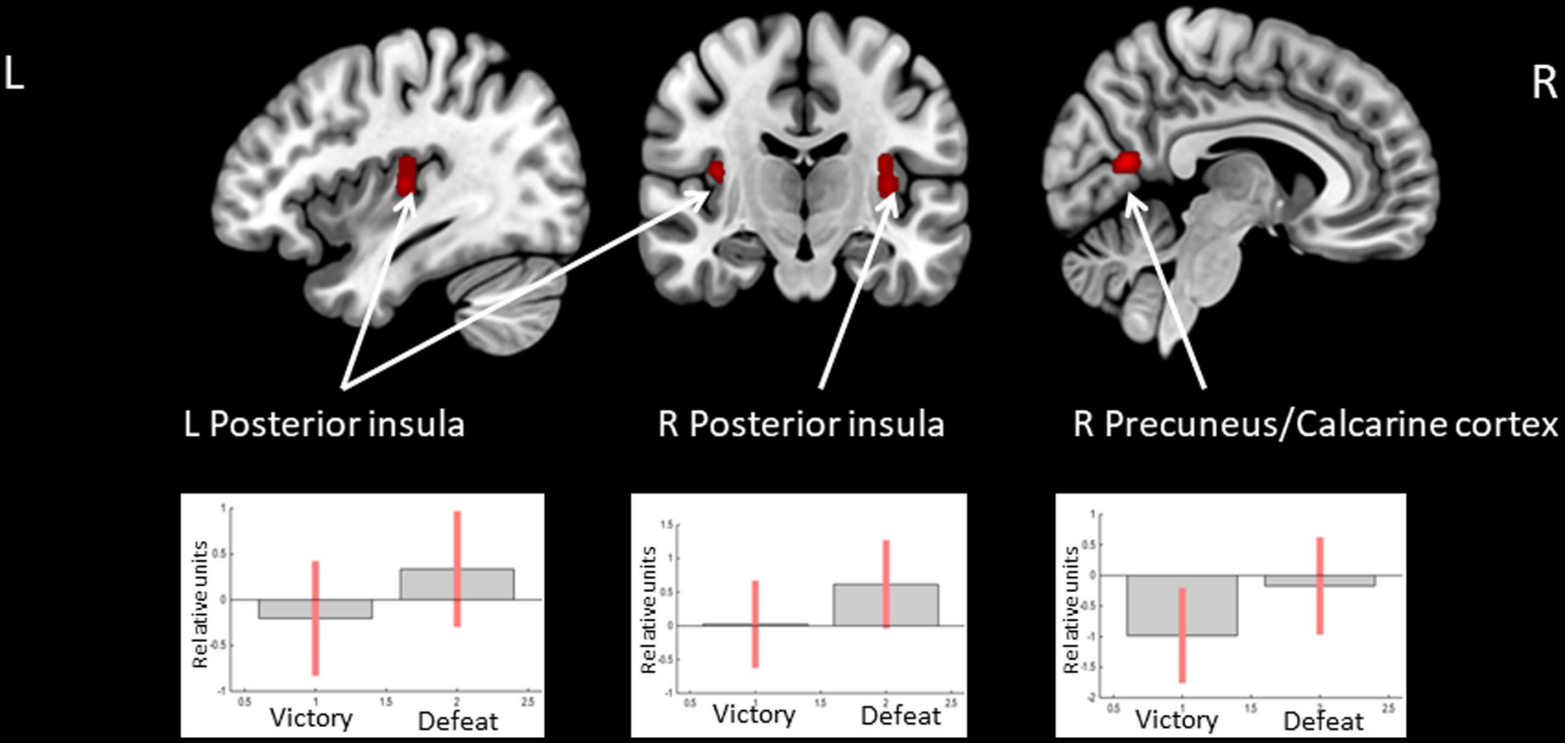
Clusters of increased BOLD signals associated with the observation of feedback informing about defeat compared to victory are colored red (t-contrast ‘defeat > victory’, log odds>3, *k* = 30). Plots show effect sizes for conditions of interest (vs. baseline) with 90% confidence intervals. R/L, right/left hemisphere.

The opposite comparison revealed increased BOLD signals in the posterior insula bilaterally and less decreased BOLD signals in the right calcarine cortex when receiving feedback, which means defeat compared to victory (see Figure 4 and Table 4).

**Table 4:** Clusters of increased BOLD signals associated with the observation of feedback that means defeat compared to victory (*t*-contrast ‘defeat> victory,’ log odds>3, *k* = 30).

**Brain region**	***k***	**Log Odds**	**Peak MNI coordinates**		
			***x***	***y***	***z***
R posterior insula	35	5.46	36	−10	7
R precuneus/calcarine cortex	39	5.25	6	−64	13
		3.63	18	−52	13
L posterior insula	36	5.12	−33	−22	16
		4.91	−36	−19	7

R/L, right/left hemisphere; k, cluster size in voxels.

### 3.4 Observation of feedback informing about victory or defeat preceding the truthful or deceitful action

**Figure 5:**
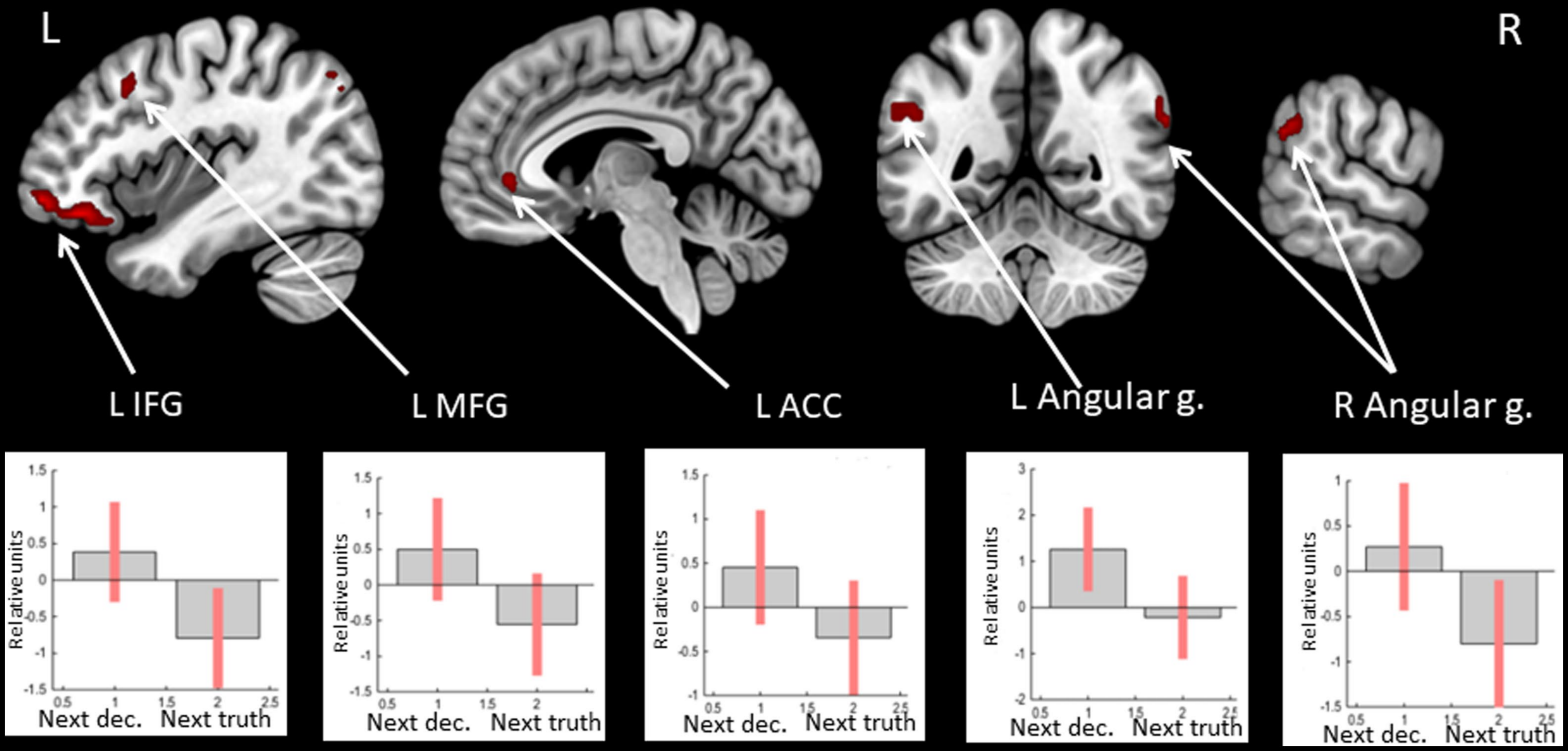
Clusters of increased BOLD signals associated with the observation of feedback informing about *victory* preceding the deceptive compared to the truthful action are colored red (t-contrast ‘next deception> next truth’ for victory condition, log odds>5, *k* = 20). Plots show effect sizes for conditions of interest (vs. baseline) with 90% confidence intervals. R/L, right/left hemisphere; g., gyrus; IFG, inferior frontal gyrus; MFG, middle frontal gyrus; ACC, anterior cingulate cortex; dec, deception.

**Figure 6:**
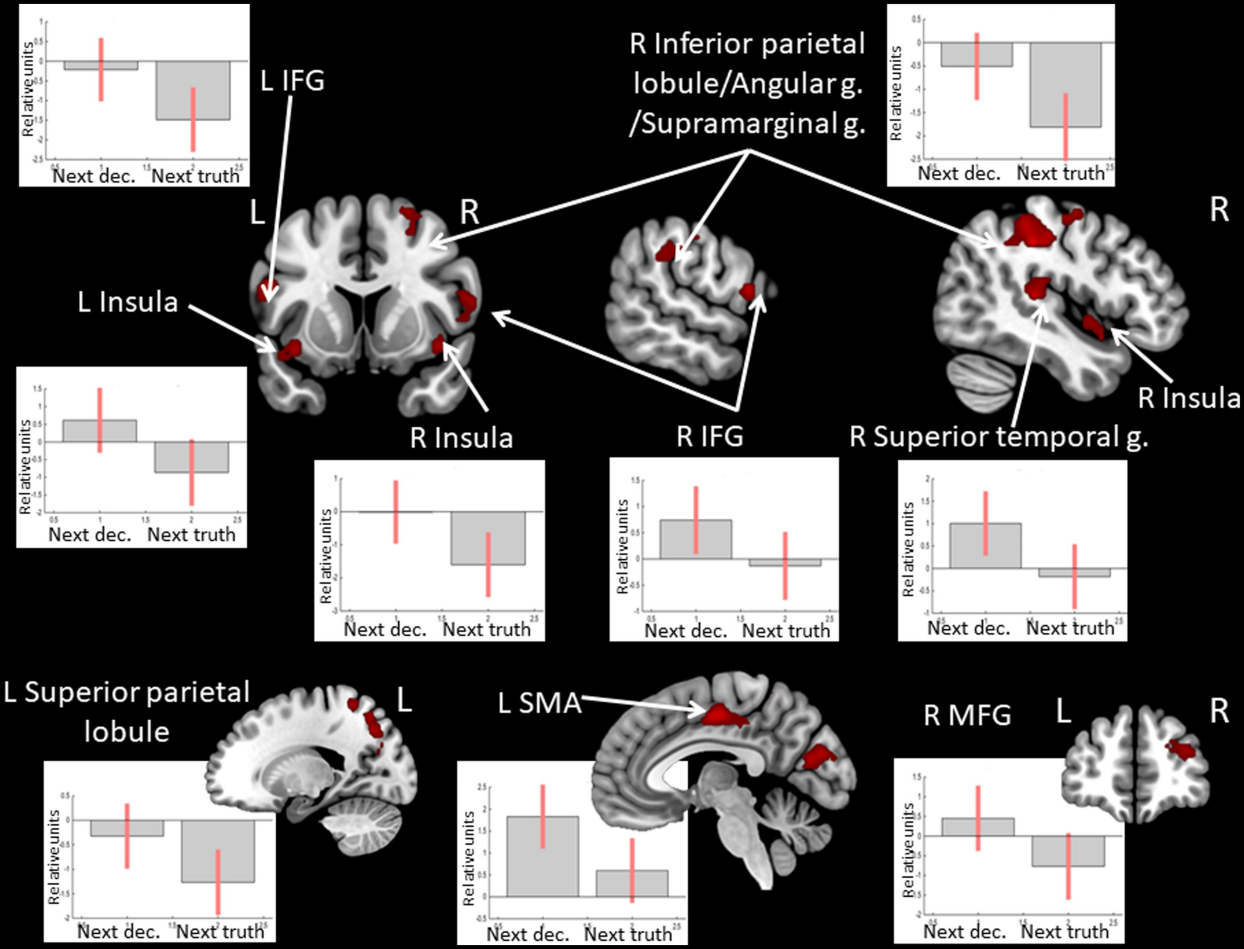
Clusters of increased BOLD signals associated with the observation of feedback informing about *defeat* preceding the deceptive compared to the truthful action are colored red (*t*-contrast ‘next deception> next truth’ for defeat condition, log odds >5, *k* = 30). Plots show effect sizes for conditions of interest (vs. baseline) with 90% confidence intervals. R/L, right/left hemisphere; g., gyrus; IFG, inferior frontal gyrus; MFG, middle frontal gyrus; SMA, supplementary motor area; dec, deception.

Observing *positive* feedback (victory) preceding the choice to deceive compared to choosing to tell the truth was associated with increased BOLD signals in the bilateral angular and supramarginal gyri, the middle frontal gyrus, the IFG, and the ACC in the left hemisphere (see Figure 5 and Table 5). The peak of the cluster in the right angular gyrus was also located in the right TPJ area, and the cluster overlapped with both its anterior and posterior subdivisions (Mars et al., 2012). On the contrary, observing *negative* feedback (defeat), preceding the choice to deceive compared to choosing to tell the truth, elicited increased BOLD signals in the IFG, the superior parietal lobule, the insula bilaterally, the inferior parietal lobule, the superior temporal gyrus, the precuneus, and the middle frontal gyrus in the right hemisphere, and the left supplementary motor area (see Figure 6 and Table 5).

**Table 5:** Clusters of increased BOLD signals associated with the observation of feedback preceding the deceptive compared to the truthful action obtained in separate analyses of victory and defeat conditions.

**Brain region**	***k***	**Log odds**	**Peak MNI coordinates**		
			***x***	***y***	***z***
***t*-contrast ‘next deception > next truth’ for victory condition, log odds > 5, *k* = 20**					
L IFG (orbital part)/insula	123	11.16	−42	35	−14
		10.00	−39	53	−8
		8.97	−24	14	−17
R angular g./supramarginal g.	47	8.26	63	−52	28
		7.91	57	−61	31
		6.86	54	−61	40
L angular g./supramarginal g.	99	8.01	−45	−70	43
		7.46	−51	−61	43
		7.36	−60	−55	28
L middle frontal g.	24	7.91	−36	17	43
L ACC	27	6.96	−6	41	1
		6.56	−12	47	7
**t-contrast ‘next deception > next truth’ for defeat condition, log odds > 5, *k* = 30**					
R inferior parietal lobule/angular g./supramarginal g.	1,017	11.06	51	−34	49
		10.56	45	−31	40
		10.28	36	−61	49
L supplementary motor area	139	9.73	−6	−16	49
		8.43	−3	−7	46
		7.17	9	−1	52
R superior temporal g.	45	9.69	45	−31	10
		6.76	45	−25	19
		5.92	48	−40	16
L Superior parietal lobule/Inferior parietal lobule/Superior occipital g.	108	8.27	−15	−67	46
		6.62	−30	−67	40
		5.86	−21	−70	34
R IFG (opercular part)	38	8.16	60	11	16
		6.38	54	14	4
L IFG (opercular part)	74	7.91	−54	11	19
		6.13	−60	−22	31
		5.68	−54	−4	28
L Superior parietal lobule	75	7.57	−24	−52	61
		6.38	−30	−43	64
		5.62	−33	−43	55
R insula	47	7.57	42	2	−5
		7.45	42	8	−11
L insula	31	7.23	−39	11	−14
R precuneus	33	7.04	12	−43	52
		5.83	9	−49	64
R middle frontal g.	41	6.89	33	50	19
		5.75	24	53	22

R/L, right/left hemisphere; g., gyrus; IFG, inferior frontal gyrus; ACC, anterior cingulate cortex; k, cluster size in voxels.

**Figure 7:**
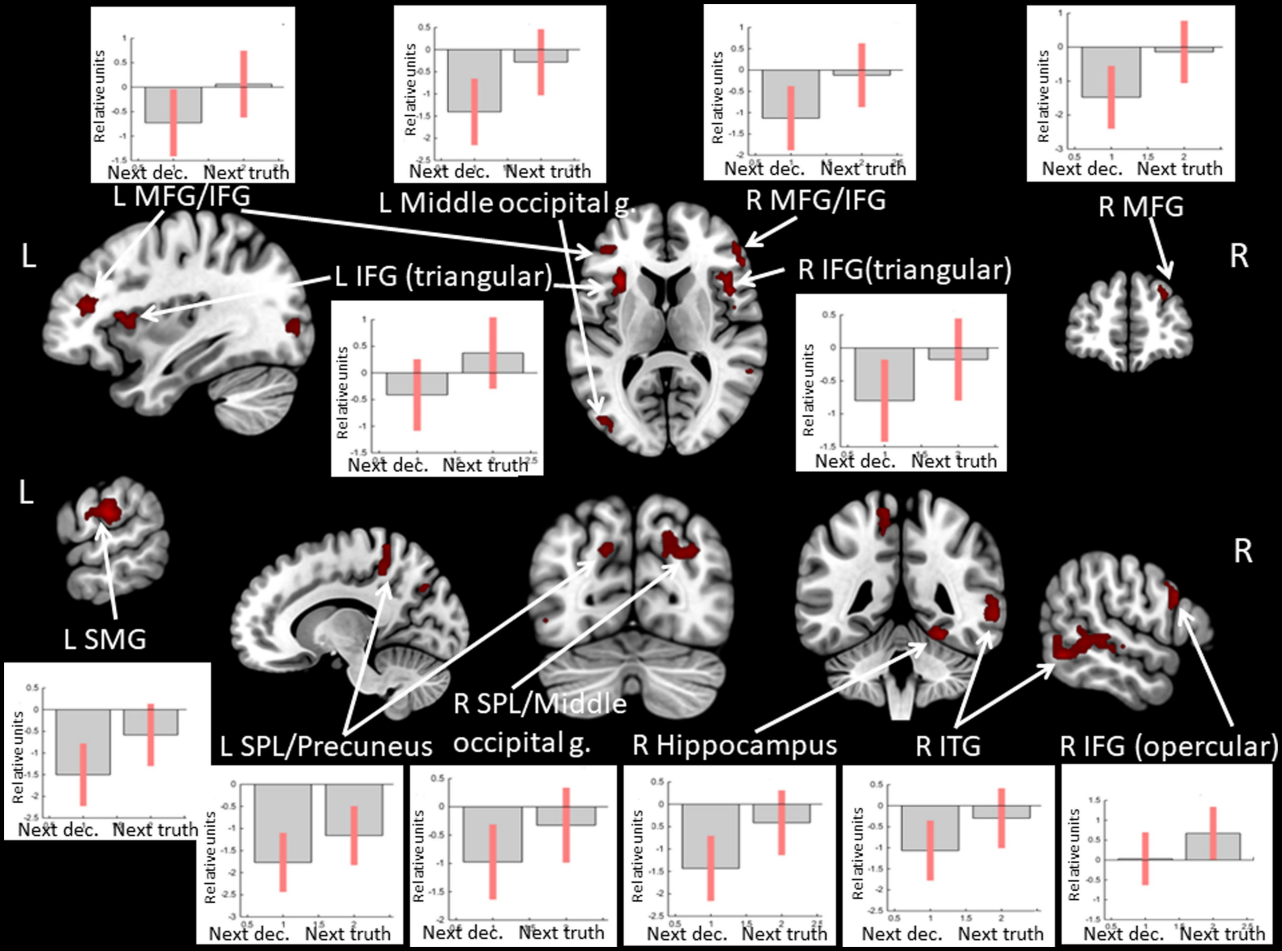
Clusters of increased BOLD signals associated with the observation of feedback informing about *victory* preceding the truthful compared to the deceitful action are colored red (*t*-contrast ‘next truth > next deception’ for defeat condition, log odds>3, *k* = 30). Plots show effect sizes for conditions of interest (vs. baseline) with 90% confidence intervals. R/L, right/left hemisphere; g., gyrus; IFG, inferior frontal gyrus; MFG, middle frontal gyrus; SMG, supramarginal gyrus; SPL, superior parietal lobule; ITG, inferior temporal gyrus; dec, deception.

Observing *positive* feedback preceding the choice to tell the truth compared to choosing to deceive was associated with increased BOLD signals in the superior parietal lobule, the middle frontal gyrus, the IFG, and the insula bilaterally, the middle occipital gyrus, the supramarginal gyrus, and the precuneus in the left hemisphere, and the inferior temporal gyrus, the fusiform gyrus, and the hippocampus in the right hemisphere (see Figure 7 and Table 6). No significant BOLD-signal changes were revealed during the observation of *negative* feedback that preceded the choice to tell the truth compared to the choice to deceive.

**Table 6:** Clusters of increased BOLD signals associated with the observation of positive feedback preceding the truthful compared to the deceitful action (t-contrast ‘next truth > next deception’ for victory condition, log odds>3, *k* = 30).

**Brain region**	***k***	**Log odds**	**Peak MNI coordinates**		
			***x***	***y***	***z***
L middle occipital g./middle temporal g./inferior temporal g.	120	7.69	−42	−85	1
		7.23	−60	−58	1
		5.02	−51	−67	−8
R hippocampus	63	7.28	21	−34	−2
		5.21	12	−25	1
		3.64	30	−22	−8
R middle frontal g./IFG	41	6.50	54	35	13
L supramarginal g.	106	6.38	−63	−25	25
		4.70	−60	−10	25
		3.91	−48	−13	19
R middle frontal g.	68	6.28	24	59	25
		5.30	15	62	25
		4.95	27	53	31
L IFG (triangular part)/ L insula	41	6.05	−33	17	10
L middle frontal g./IFG	90	5.75	−36	35	16
		5.31	−51	29	25
		3.96	−45	44	19
R fusiform g.	35	5.68	27	−46	−17
	52	5.39	−21	−61	−8
R IFG (triangular part)/R insula	53	5.34	45	11	10
		3.79	48	2	7
L superior parietal lobule/precuneus	42	5.15	−21	−64	22
		4.79	−21	−67	43
		4.42	−15	−70	34
R inferior temporal g.	172	5.10	57	−55	−8
		4.86	60	−43	−2
		4.63	48	−55	4
R IFG (opercular part)	35	4.87	60	11	31
		4.59	51	29	31
L precuneus	35	4.81	−15	−40	46
		4.17	−12	−46	67
		3.58	−9	−43	58
R superior parietal lobule/middle occipital g.	51	4.46	27	−70	31
		3.92	21	−73	43
		3.92	18	−67	49

R/L, right/left hemisphere; g., gyrus; IFG, inferior frontal gyrus; k, cluster size in voxels.

## 4 Discussion

The current study was dedicated to investigating the neural mechanisms underlying the processing of opponent feedback in relation to consequent deceptive behavior. The results obtained indicate that feedback presentation prior to deception, compared to forthcoming honest action, is associated with increased BOLD signals in the left ACC and the left anterior insula and suppressed BOLD signals in the right hippocampus. On the contrary, the observation of feedback preceding the true versus deceitful response is associated with suppressed BOLD signals in the right TPJ and the left IFG. As the rate of consequent deception (vs. truth) did not depend on feedback type, the observed differences in brain activity are associated with the following action preparation and not the feedback itself. These results are in line with the results obtained previously for the moment of execution of the deceptive action. It was found that local activity in the bilateral supramarginal and angular gyri (including the right TPJ), and the left IFG was increased when the deception compared to truth was chosen in the forthcoming trial only during the evaluation of positive feedback. These observations corroborate the hypothesis that feedback valence influences BOLD signals associated with the choice of forthcoming deception versus truth.

### 4.1 Observing feedback during the task of manipulation by deception recruits brain mechanisms related to the choice of deception

We demonstrated an increase in the BOLD signal in the left ACC and the left anterior insula preceding deceptive actions, which is in line with existing knowledge of brain mechanisms of deception previously shown to be involved during the time of selection of deceptive responses. In particular, meta-analytic studies emphasize the involvement of the ACC in the performance of deception (Christ et al., 2009; Lisofsky et al., 2014; Delgado-Herrera et al., 2021). Its functional role during deception is believed to be related to performance monitoring and can reflect conflict resolution (Abe et al., 2018), considering the potential risk of social confrontation (Sip et al., 2012), and detection of the incompatibility of deceptive choice and known truthful information named error detection mechanism (Bechtereva and Gretchin, 1969; Bechtereva et al., 2005; Kireev et al., 2013, 2019). Importantly, ACC was specifically involved in the performance of deception in tasks with high ecological validity (Delgado-Herrera et al., 2021) and in a social interactive context (Lisofsky et al., 2014), creating conditions for an increased requirement for performance monitoring. The role of the anterior insula in deception may be in coding the influence of intentions of deceptive actions. For example, increased activity in the right insula characterized deception with intention compared to false memories (Yu et al., 2019), while altruistic goals of lying compared to self-serving goals reduced activity in the anterior insula (Yin et al., 2017).

Interestingly, it was also observed that the BOLD signal in the right hippocampus was suppressed in conditions under which a deceptive action was chosen in the forthcoming trial. Reduced activity in hippocampi has previously been reported during effortful retrieval, memory search, and suppression-induced forgetting of unwanted memories (Reas et al., 2011; Reas and Brewer, 2013; Gagnepain et al., 2017; Apšvalka et al., 2020). It was hypothesized to be the mechanism of suppression of non-selected solutions in tasks for the completion of ambiguous, fragmented phrases, which may rely on neurophysiological mechanisms of inhibitory control (Kireev et al., 2022). Deception is associated with a higher cognitive load than truth-telling. One possible explanation is the involvement of the process of inhibition of the default and dominant truthful response prior to performing deception (Lee et al., 2010; Gamer and Ambach, 2014; Debey et al., 2015). It was previously associated with increased activity in the frontal and parietal regions (Ito et al., 2011; Farah et al., 2014; Ofen et al., 2017). Therefore, suppressed activity in the hippocampus prior to deception may reflect the process of suppression of non-selected truth. The result obtained supports the notion of the increased requirement for inhibition associated with deception and extends the list of areas associated with this process.

Taken together, the obtained experimental data suggest that observing the feedback during the task on deception recruits not only the mechanisms of processing the result but also the mechanisms of performance monitoring, intention assessment, and suppression of non-selected solutions connected to the forthcoming action. These processes are especially important for deliberate deception. Intentionality is believed to be one of the key features of deceptive behavior in real life. However, earlier neuroimaging studies used tasks that explicitly instructed participants in which trials they were supposed to lie and in which—to tell the truth (e.g., Christ et al., 2009). More ecological settings like the one used in the current study allow participants to choose between deception and truth in each game trial (Kireev et al., 2007; Greene and Paxton, 2009). Earlier neurophysiological results of these studies indicated that deliberate deception requires relatively greater involvement of both higher-level processes such as cognitive control, monitoring, evaluating potential risks and benefits, and assessing the opponent’s mental state (for review, see Sip et al., 2008), as well as greater emotional load (Yin et al., 2016). Findings in the current study add substantially to present knowledge on possible neural mechanisms of deception, including their participation prior to action execution, during the time of feedback evaluation preceding deception.

Alternatively, BOLD-signal changes in the ACC and anterior insula may reflect the influence mechanism of reward processing on future action selection via reinforcement learning. Activity in the left anterior insula during feedback is argued to be directly modulated by the dorsal striatum (Menon and Levitin, 2005) and was correlated with both the surprise and the valence components of the reward prediction error (RPE) (Fouragnan et al., 2018). ACC, in turn, is suggested to be responsible for the process of selecting and maintaining high-level options that contribute to reinforcement learning during goal-directed behavior (Holroyd and Yeung, 2012; Hu et al., 2015). Therefore, increased BOLD signals in the ACC and anterior insula when observing feedback before selecting deception (vs. truth) possibly reflect the increased requirement of behavioral adjustment via reinforcement learning.

### 4.2 Observing feedback during the task of manipulation by deception recruits brain mechanisms related to the choice of truth

The suppressed activity in the right TPJ and the left IFG during feedback observation prior to performing the truthful action compared to the deceitful action suggests a possible role of focusing attention on the stimulus to truthfully report it to the opponent. A TPJ is a region in the ventral part of the inferior parietal lobule at the intersection of the supramarginal gyrus, the angular gyrus, and the posterior superior temporal. It was associated with functions including stimulus-driven attention and mental state attribution (Saxe, 2006; Corbetta et al., 2008). Namely, the anterior right TPJ is part of the ventral attention network (VAN), which also includes the right IFG and allows the orientation of the attention to unattended, relevant, or salient stimuli (Corbetta and Shulman, 2002; Corbetta et al., 2008; Vossel et al., 2014). However, some research points out that it is not exclusively right-lateralized: the left TPJ and the left IFG responded to the contextual relevance of nontarget stimuli (DiQuattro and Geng, 2011). Importantly, fMRI studies that focused on the period of stimulus anticipation revealed that the right TPJ activity is suppressed during tasks that require high short-term memory and focused top-down attention (Shulman et al., 2003, 2007; Todd et al., 2005; Solís-Vivanco et al., 2021). Furthermore, the deactivation in the right TPJ increased with increasing short-term memory load (Todd et al., 2005). Furthermore, stronger functional inhibition of the VAN during relevant stimulus anticipation was associated with better task performance (Shulman et al., 2003; Solís-Vivanco et al., 2021). Taken together, these findings suggest that when planning to tell the truth the processes of preventing attention shift from the stimulus and protection from distractors may move at the forefront, manifesting in suppressed activity in the areas of the VAN network.

### 4.3 Feedback valence during feedback observation in the task of manipulation by deception influences the local activity in reward-related brain areas

The results indicate that the activity in the CN bilaterally is suppressed while observing unsuccessful compared to successful outcomes for both deceptive and honest actions. In turn, feedback that informs defeat compared to victory is associated with increased activity in the bilateral posterior insula. The results obtained are consistent with the current knowledge of reward-related brain areas and confirm that the model used for data analysis is appropriate.

BOLD signals in the CN are linked to the RPE mechanism, which plays a role in learning by representing the difference between expected and obtained rewards. Research on reward processing has demonstrated that midbrain dopamine neurons, particularly in the ventral tegmental area and substantia nigra encode RPE (Schultz et al., 1997; Bayer and Glimcher, 2005; Lak et al., 2014). When the reward is less than predicted, the firing rate of midbrain dopamine neurons will temporarily drop, generating a negative RPE (Schultz, 2002). BOLD signal in the CN, which receives projections from the substantia nigra, also correlates with the RPE (Delgado et al., 2000; O’Doherty et al., 2004; Haruno and Kawato, 2006; Burke et al., 2010; Cooper et al., 2012; Fouragnan et al., 2013, 2018). Delgado et al. (2000) demonstrated that activity in the CN falls below baseline when receiving monetary punishment. Non-monetary prediction errors were also linked to CN deactivation. That is, violation of trust from cooperative opponents elicited stronger CN deactivation when participants had positive prior beliefs about the opponent than when they did not have them (Fouragnan et al., 2013). Similar deactivation of the CN was shown when participants observed how another person received an unexpectedly low reward (Cooper et al., 2012). In our task, the goal was to select options leading to reward; thus, loss and monetary punishment were associated with a lower-than-expected result and, possibly, negative RPE, reflected by suppressed bilateral CN activity.

The RPE signal includes two components: first, connected to a surprise (a degree of unexpectedness), and second, connected to a valence (positive or negative) (Schultz, 2016). The activity in the CN reflected the surprise component of RPE (Fouragnan et al., 2018). Our results are in line with this because all RPE in our task had negative valence, and only the surprise component was changing.

In turn, the increased response to defeat compared to victory in the bilateral posterior insula is consistent with its implications in assessing feedback valence (Tricomi et al., 2006; Bischoff-Grethe et al., 2009; Luking and Barch, 2013; Becker et al., 2014), risk-taking (Canessa et al., 2011), perceiving angry or aversive stimuli (Straube et al., 2004; Simmons et al., 2006; Paulus et al., 2010; Mazzola et al., 2016). However, in other studies, comparatively increased activity was demonstrated in the posterior insula for loss (Tricomi et al., 2006), reward (Luking and Barch, 2013), or uninformative/neutral feedback (Bischoff-Grethe et al., 2009). Interestingly, the study that, in line with our results, reported greater activation to loss, compared to reward, also used monetary reward (compared to non-monetary, e.g., candies or showing scores) (Tricomi et al., 2006). Thus, the posterior insula might be sensitive not only to the feedback valence but also to its monetary or non-monetary type.

In general, these results suggest that the areas associated with reward evaluation, including the RPE mechanism, change their activity during positive and negative feedback evaluation in tasks that allow deception in accordance with existing knowledge on changes in reward-related brain activity.

### 4.4 Feedback valence during feedback observation in the task of manipulation by deception influences the local activity in brain areas related to the choice

The most striking observation to emerge from the analysis was that only during the evaluation of positive feedback was the local activity in the bilateral TPJ, and the left IFG increased when the deception was chosen in the forthcoming trial. Furthermore, activity in the same areas in the bilateral superior parietal lobule, the left supramarginal gyrus, and the left precuneus was suppressed more prior to deceiving compared to telling the truth when the feedback was positive and prior to telling the truth than deceiving when it was negative. Furthermore, local activity in the right IFG prior to deceiving compared to telling the truth increased when the feedback was negative but was more suppressed when the feedback was positive. These results corroborate that the feedback valence influences BOLD signals in the frontal and parietal areas, which are associated with the choice of forthcoming deception versus truth.

Along with the role of attention reorienting, the TPJ participates in stimulus evaluation. It is argued to be activated in a sustained manner during the computation of the behavioral significance of the stimulus by comparing it with an internal representation (Bae et al., 2021). The other role of TPJ (posterior subdivision)is connected to the so-called theory of mind ability associated with the attribution of thoughts, intentions, and beliefs to an opponent. It was argued that attention and social cognition share computational properties in the right TPJ (Corbetta et al., 2008). Specifically, the reorienting of attention and false belief tasks overlap in the anterior right TPJ according to the conjunction meta-analysis (Krall et al., 2015). For deception, specifically, the theory of mind involvement is especially relevant (Sip et al., 2008). Local activity and functional connectivity in areas of the theory of mind network, including the bilateral TPJ, were previously shown to increase during the choice to deceive compared to the choice to tell the truth (Lisofsky et al., 2014; Volz et al., 2015; Zheltyakova et al., 2020, 2022). It is possible to speculate that deactivation of the right TPJ prior to telling the truth is associated with preventing the attention shift, while its activation prior to deceiving can signify the key role of social cognition in interpreting feedback prior to performing deception. However, further research is required to support this assumption. Concerning the stage of feedback observation, local activity in the right TPJ during feedback processing reflects neural computations consisting of estimating beliefs about how participant’s actions influence their opponent’s strategy and updating these beliefs (Hampton et al., 2008). These computations influence strategic decisions through system-level interaction within the theory of mind network (Hill et al., 2017). Furthermore, disrupting neural excitability in the right TPJ reduces the neural indices of these computations and the functional connectivity of the right TPJ and makes behavior more predictive (Hill et al., 2017).

Taken together, these findings suggest that negative feedback evaluation in tasks that allow deception is associated with mechanisms of processing information about defeat and with negative RPE. It hypothetically influences the involvement of areas associated with the choice of subsequent action. Conversely, when processing information about victory in the absence of negative RPE-related suppression, the forthcoming selection of deception (vs. truth) requires additional effort associated with stimulus evaluation and social-cognitive processes, including mentalizing-related neural computations. The current study emphasizes the importance of further research on the influence of reward-related brain mechanisms on the local activity of areas associated with the choice of deception versus truth during feedback observation.

## 5 Conclusion

In conclusion, the current article has revealed local BOLD-signal changes that underlie the processes of preparation for deception, that is, during the presentation of the opponent’s feedback to a previous action. It was shown that several brain regions that were previously demonstrated as involved in deception execution, such as the left ACC and anterior insula, also underlie processes related to deception preparation. The obtained results allowed us to suggest that these processes may include performance monitoring, intention assessment, suppression of non-selected solutions, and the influence of reward processing on future action selection via reinforcement learning. These results broaden the understanding of brain mechanisms underlying deception and highlight the importance of studying brain mechanisms underlying the choice of deception versus truth at different stages of decision-making.

## Data availability statement

The raw data supporting the conclusions of this article will be made available by the authors, without undue reservation.

## Ethics statement

The studies involving humans were approved by the Ethics Committee of the N.P. Bechtereva Institute of the Human Brain of the Russian Academy of Sciences. The studies were conducted in accordance with the local legislation and institutional requirements. The participants provided their written informed consent to participate in this study.

## Author contributions

MZ: Conceptualization, Formal analysis, Writing – original draft, Writing – review & editing. AK: Conceptualization, Investigation, Supervision, Writing – original draft, Writing – review & editing. DC: Conceptualization, Funding acquisition, Project administration, Resources, Writing – review & editing. MD: Conceptualization, Funding acquisition, Resources, Supervision, Writing – review & editing. MK: Conceptualization, Formal analysis, Investigation, Methodology, Project administration, Supervision, Validation, Writing – original draft, Writing – review & editing.

## Footnotes

^1^ http://www.fil.ion.ucl.ac.uk/spm

^2^ https://www.alivelearn.net/xjview/

^3^ https://www.nitrc.org/projects/mricrogl/
